# SlicerCBM: automatic framework for biomechanical analysis of the brain

**DOI:** 10.1007/s11548-023-02881-7

**Published:** 2023-04-01

**Authors:** Saima Safdar, Benjamin F. Zwick, Yue Yu, George C. Bourantas, Grand R. Joldes, Simon K. Warfield, Damon E. Hyde, Sarah Frisken, Tina Kapur, Ron Kikinis, Alexandra Golby, Arya Nabavi, Adam Wittek, Karol Miller

**Affiliations:** 1 Intelligent Systems for Medicine Laboratory, The University of Western Australia, 35 Stirling Highway, Perth, WA Australia; 2 Computational Radiology Laboratory, Boston Children’s Hospital, Boston, MA USA; 3 Brigham and Women’s Hospital, Boston, MA USA; 4 Harvard Medical School, Boston, MA USA; 5 Department of Neurosurgery, KRH Klinikum Nordstadt, Hannover, Germany; 6 Department of Agriculture, University of Patras Nea Ktiria, 30200 Campus Mesologhi, Greece

**Keywords:** Brain deformation, Biomechanics, Brain shift, Framework

## Abstract

**Purpose:**

Brain shift that occurs during neurosurgery disturbs the brain’s anatomy. Prediction of the brain shift is essential for accurate localisation of the surgical target. Biomechanical models have been envisaged as a possible tool for such predictions. In this study, we created a framework to automate the workflow for predicting intra-operative brain deformations.

**Methods:**

We created our framework by uniquely combining our meshless total Lagrangian explicit dynamics (MTLED) algorithm for computing soft tissue deformations, open-source software libraries and built-in functions within 3D Slicer, an open-source software package widely used for medical research. Our framework generates the biomechanical brain model from the pre-operative MRI, computes brain deformation using MTLED and outputs results in the form of predicted warped intra-operative MRI.

**Results:**

Our framework is used to solve three different neurosurgical brain shift scenarios: craniotomy, tumour resection and electrode placement. We evaluated our framework using nine patients. The average time to construct a patient-specific brain biomechanical model was 3 min, and that to compute deformations ranged from 13 to 23 min. We performed a qualitative evaluation by comparing our predicted intra-operative MRI with the actual intra-operative MRI. For quantitative evaluation, we computed Hausdorff distances between predicted and actual intra-operative ventricle surfaces. For patients with craniotomy and tumour resection, approximately 95% of the nodes on the ventricle surfaces are within two times the original in-plane resolution of the actual surface determined from the intra-operative MRI.

**Conclusion:**

Our framework provides a broader application of existing solution methods not only in research but also in clinics. We successfully demonstrated the application of our framework by predicting intra-operative deformations in nine patients undergoing neurosurgical procedures.

## Introduction

During neurosurgery, the brain undergoes significant deformation known as brain shift, making it challenging to precisely locate the surgical target, such as a tumour or epileptic seizure onset zone. Intra-operative magnetic resonance images (MRIs) can provide the location of the surgical target during neurosurgery. However, the cost of intra-operative magnetic resonance imaging scanners is high (over $10 million) [1] and brain MRI acquisition takes a long time (about 45 to 60 min) [2], which interferes with the surgical operation. Furthermore, intra-operative MRI cannot be acquired for patients with electrodes implanted within the brain in epilepsy surgery.

Brain shift, which refers to the significant deformation of the brain during neurosurgery, can be analysed in purely mechanical terms using established methods of continuum mechanics [3]. To solve the equations of continuum solid mechanics, suites of computational biomechanics finite element and meshless algorithms [4, 5] exist to predict organ deformation, including brain deformations. An example is a suite of meshless total Lagrangian explicit dynamics (MTLED) algorithms based on the total Lagrangian formulation of nonlinear solid mechanics and explicit time domain integration [5–7] developed by our research group (Intelligent Systems for Medicine Lab). The MTLED algorithm has been extensively evaluated in previous studies for computing soft tissue deformations [7–9]. However, it is very sophisticated and requires specialised knowledge of computational biomechanics and numerical methods to set up a simulation. MTLED uses a cloud of points to discretise the problem domain. It is comparatively easy to generate a biomechanical model with a cloud of points rather than a high-quality finite element mesh but defining boundary conditions and loading and assigning material properties to intra-cranial constituents are still required.

In this study, we created a framework to automate the workflow for generating a patient-specific brain biomechanical model and computing the intra-operative deformations using the MTLED algorithm. We implemented our framework as an extension, SlicerCBM (Computational Biophysics for Medicine in 3D Slicer), for the 3D Slicer medical imaging platform [10]. SlicerCBM is freely available from our GitHub repository (https://github.com/SlicerCBM/SlicerCBM). The framework computes brain deformations for three different neurosurgical brain shift scenarios: craniotomy-induced brain shift (due to opening of the skull), tumour resection-induced brain shift (due to removal of the tumour) and electrode placement-induced brain shift (due to placement of electrocorticography electrodes on the brain surface after craniotomy in epilepsy surgery). We evaluate our framework for predicting brain deformations for nine patients (Table 1) undergoing three different neurosurgical brain shift scenarios. The data for this study were obtained from the databases of the Surgical Planning Laboratory (SPL) at Brigham and Women’s Hospital, Computational Radiology Laboratory (CRL) at Boston Children’s Hospital and Montreal Neurological Institute’s Brain Images of Tumours for Evaluation [11].

**Table 1:** Pre-operative (pre-op), intra-operative (intra-op) and post-operative (post-op) patient data analysed in this study

Case	Application	3D image data type	Slice thickness (mm)
1	Craniotomy	Pre-op MRI and intra-op MRI	2.5
2	Craniotomy	Pre-op MRI and intra-op MRI	2.5
3	Craniotomy	Pre-op MRI and intra-op MRI	2.5
4	Tumour resection	Pre-op MRI and intra-op MRI	2.2
5	Tumour resection	Pre-op MRI and post-op MRI	4.0
6	Tumour resection	Pre-op MRI and post-op MRI	2.0
7	Tumour resection	Pre-op MRI and post-op MRI	2.0
8	Electrode placement	Pre-op MRI and post-op CT with electrodes implanted	0.7
9	Electrode placement	Pre-op MRI and post-op CT with electrodes implanted	1.0

## Methods

**Fig. 1:**
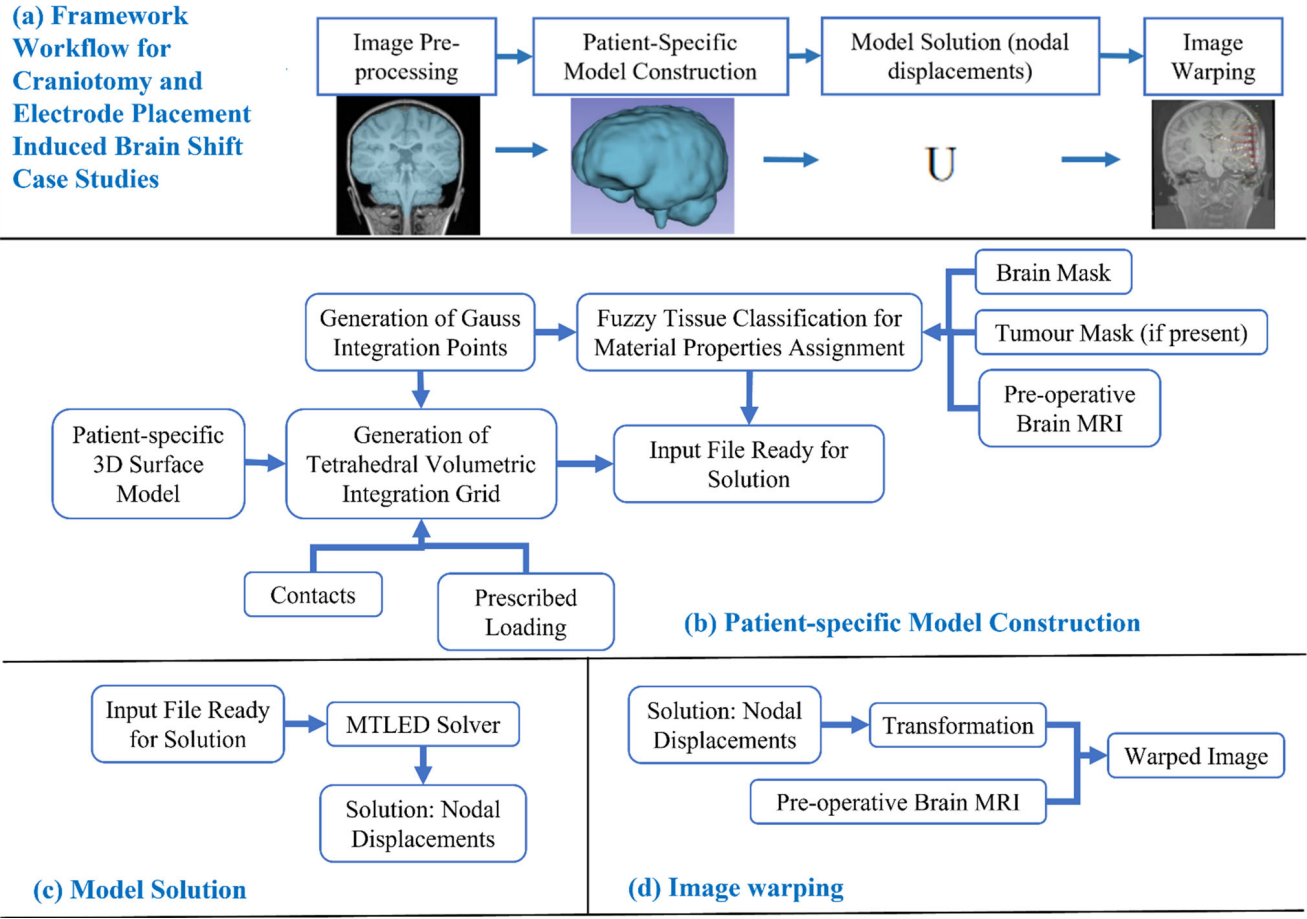
Workflow of our framework for generating and solving a patient-specific model of craniotomy and electrode placement-induced brain shift scenarios, **a** craniotomy and electrode placement-induced brain shift, **b** patient-specific computational grid generation, **c** model solution using meshless total Lagrangian explicit dynamic (MTLED) algorithm and **d** image warping

**Fig. 2:**
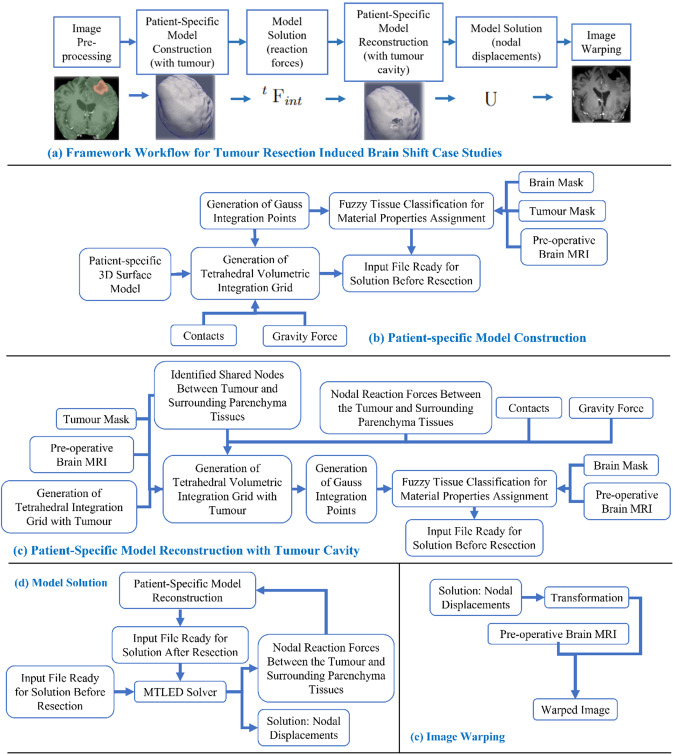
Workflow of our framework for generating and solving a patient-specific model of tumour resection-induced brain shift scenarios, **a** tumour resection-induced brain shift, **b** patient-specific computational grid generation, **c** patient-specific computational grid generation (with tumour cavity), **d** model solution using meshless total Lagrangian explicit dynamic (MTLED) algorithm and **e** image warping

Figure 1 describes the workflow of our framework for craniotomy-induced and electrode placement-induced brain shift, whereas Fig. 2 describes the workflow for tumour resection-induced brain shift. The developed framework modules corresponding to each component of the framework are discussed in Sects. "Patient-specific biomechanical model generation" to "Image warping ".

### Image pre-processing

We used the rigid registration algorithm [12] in the “GeneralRegistration” module of 3D Slicer to obtain the pre-operative brain anatomy in the intra-operative brain orientation. We automatically extracted the brain parenchyma (known as skull stripping) from the high-quality rigidly registered pre-operative MRI using the watershed algorithm of FreeSurfer (http://surfer.nmr.mgh.harvard.ed), an open-source software suite for analysing medical resonance images (MRIs) [13]. Following skull stripping, the cropped pre-operative MRI contains only the brain tissues, tumour and ventricles. To segment the tumours, we used the “GrowfromSeeds” feature of 3D Slicer’s built-in module “SegmentEditor”, which utilises the “FastGrowCut” algorithm to generate a tumour mask [10]. Generation of this tumour mask is automatic but may require corrections by an analyst. We used the tumour mask to locate the nodes and integration cells that represent a tumour within the brain (see Sect. "Computational grid generator").

### Patient-specific biomechanical model generation

#### Computational grid generator

**Fig. 3:**
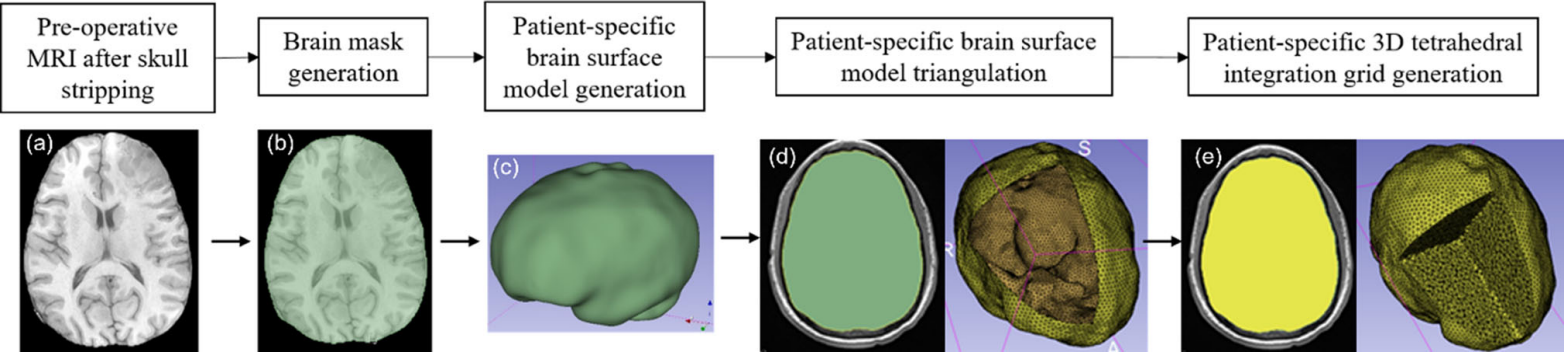
Workflow for automatic patient-specific brain integration grid generation within our 3D Slicer module **a** pre-operative MRI after skull stripping, **b** brain mask generation using threshold, **c** brain surface model generation using marching cubes algorithm, **d** brain surface model triangulation (yellow) using Voronoi clustering algorithm (yellow line around brain surface model (green) represents the brain triangulation) and **e** 3D tetrahedral integration grid (yellow) generation using 3D Delaunay algorithm

To discretise the problem domain, which is the brain parenchyma extracted from the pre-operative MRI, we used a cloud of points. We developed a patient-specific tetrahedral integration grid using our "ComputationalGridGenerator" module, which takes the cropped pre-operative MRI as input and automatically generates the integration grid (Figs. 2 and 3).

The procedure implemented in our "ComputationalGridGenerator" module involves the following steps: first, it takes the pre-operative MRI after skull stripping and generates a brain mask using Kittler-Illingworth [14] thresholding algorithm. Next, it generates a brain surface model using the marching cubes algorithm [15]. Then, it generates a uniformly triangulated brain surface using the Voronoi clustering algorithm [16] of PyACVD (https://github.com/pyvista/pyacvd). Finally, it generates a tetrahedral grid using the 3D Delaunay algorithm of Gmsh [17]. To smooth the brain surface, we used the Laplacian filter [18] (Fig. 2). It is crucial to understand that the tetrahedral integration cells are not finite elements and do not have to adhere to the strict quality requirements of a finite element mesh. Table 2 lists the number of nodes, integration cells and integration points generated using our “ComputationalGridGenerator” module for all nine patients.

**Table 2:** Summary of computational grids generated with respect to the patient-specific brain

Case	No. of nodes	No. of integration cells	No. of integration points
1	33,273	141,935	567,740
2	40,767	169,026	676,104
3	49,195	210,196	840,784
4	22,507	119,170	119,170
5	24,675	129,486	129,486
6	26,189	136,825	136,825
7	23,961	126,417	126,417
8	33,363	136,477	545,908
9	21,788	55,470	221,880

**Fig. 4:**
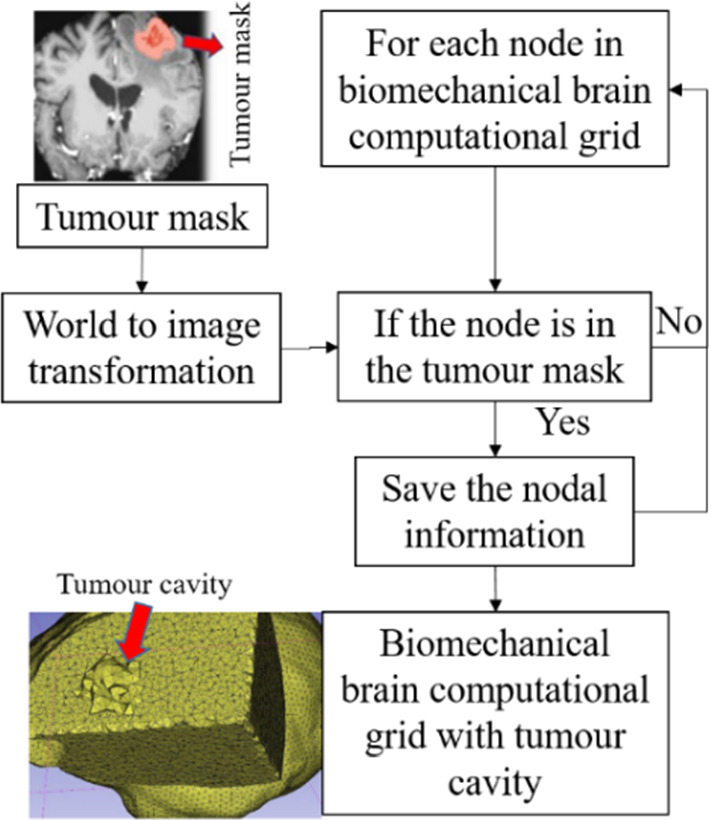
Procedure for generating a brain computational grid with tumour cavity as implemented in our module “TumourResectionAndBrainRemodelling”

To predict the tumour resection-induced brain shift, a computational grid of the brain with a tumour cavity is required. This grid is used to apply traction forces at the boundary of the tumour cavity, as described in our previous study [9]. To automate the construction of a brain computational grid with a tumour cavity, we developed the "TumourResectionAndBrainRemodelling" module (shown in Fig. 4). This module takes the brain computational grid and the tumour mask as inputs, and identifies the nodes within the tumour mask to generate a brain computational grid with a tumour cavity. The coordinates of these identified nodes are saved and used to construct the new brain computational grid with the tumour cavity.

#### Brain–skull contact interface

To account for the difference in stiffness between the skull and brain tissues, a frictionless sliding contact interface is defined between the rigid skull surface and the deformable brain model in neurosurgical brain shift scenarios. This approach prevents brain nodes from penetrating the skull while allowing the brain to slide along the inner surface of the skull [19].

To define the contacts automatically, we developed our module “SkullGenerator” to construct a skull surface model using the triangulated brain surface model generated in Sect. "Computational grid generator"; and to extract the brain surface nodes in contact with the skull surface model.

#### Loading

**Fig. 5:**
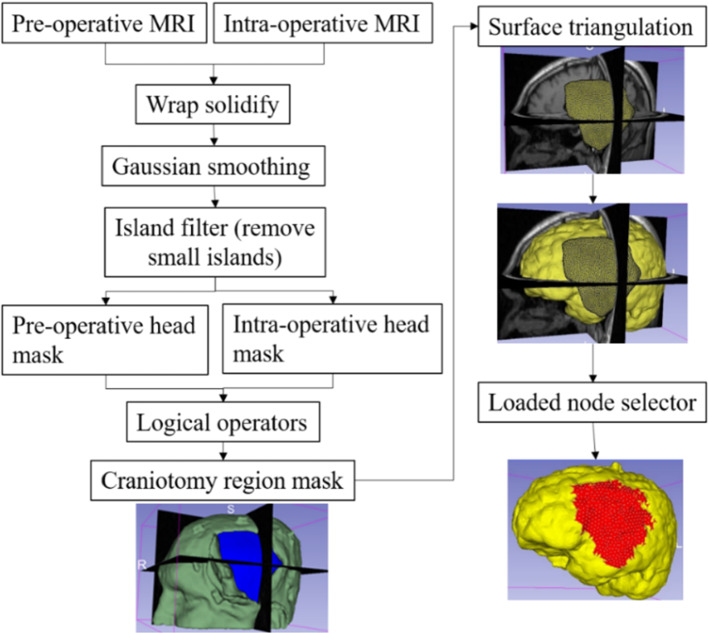
Procedure for automatically creating a craniotomy region and selecting brain surface nodes exposed due to craniotomy

In craniotomy-induced and electrode placement-induced brain shift, loading is defined as prescribed displacements on the exposed part of the brain due to neurosurgical procedures. In tumour resection-induced brain shift, loading is defined as gravity forces. To define the load, information about the deformation of the exposed brain surface can be obtained using cameras or the pointing tool of a neurosurgical station [20]. In this study, we acquire such information using sparse intra-operative MRI. To define the prescribed loading, selection of the exposed surface area of the brain is an essential step. Our automated procedure for selecting brain surface nodes exposed due to craniotomy is implemented in the modules “CranGenerator” and “NodeSelector”, which streamlines the process of identifying loaded nodes. It comprises the following steps (Fig. 5): (1) auto-thresholding is used to select the patient’s head in pre-/intra-operative MRIs to create a pre-/intra-operative head mask, (2) wrap solidify effect is used to shrink wrap [10] and remove any gaps in pre-/intra-operative head masks generated in step 1, (3) Gaussian smoothing (3 mm) is used to smooth the created head masks, and Island filter [10] is used to remove small islands (1000 voxels), (4) logical operator (subtract) is used to create a craniotomy region mask, (5) marching cubes algorithm [15] is used to generate the craniotomy surface model, and (6) brain surface cells exposed due to craniotomy are selected using the craniotomy surface model from step 5, and finally, the brain surface nodes are selected.

To compute the prescribed displacements for the selected brain surface nodes, we use a procedure that involves several steps. Firstly, we extract sparse pre-operative and intra-operative MRI information, which we then use to compute the B-Spline transform using a rigid registration algorithm [12]. Secondly, we apply the B-Spline transform to the selected brain surface nodes that are exposed due to craniotomy, resulting in the position of the brain surface nodes in an intra-operative (deformed) brain configuration. Finally, we compute the prescribed displacements by calculating the difference between the coordinates of the brain surface nodes in the deformed brain configuration and those in the undeformed configuration. This procedure enables us to accurately determine the necessary displacement values for the nodes on the brain surface, which is crucial for simulating the deformation of the brain during neurosurgery.

**Fig. 6:**
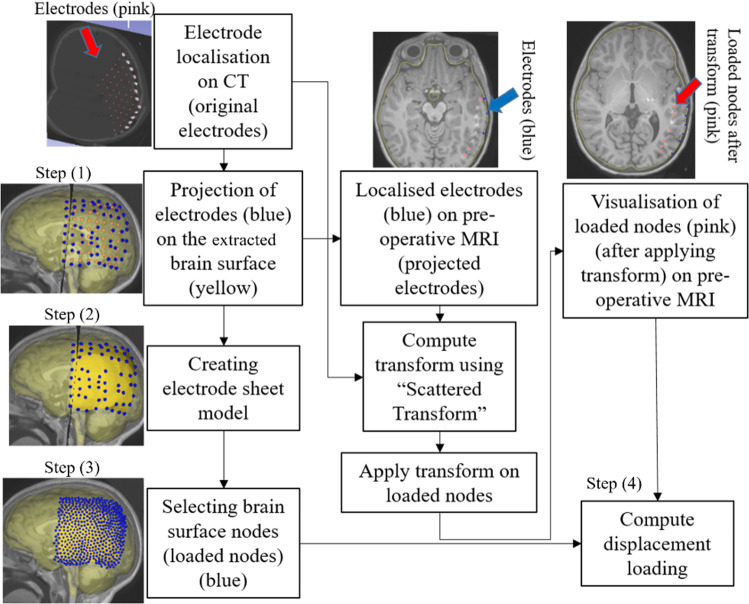
Procedure for selecting brain surface nodes under electrode sheet and computing the prescribed displacements

For electrode placement-induced brain shift, we extracted the electrode locations (coordinates) from the computed tomography (CT) image using our electrode extraction procedure implemented as “ElectrodesToMarkups” module. The steps involved in our electrode extraction procedure are: (1) creating a binary label volume from binary CT image using PolySeg software library, (2) splitting the binary label volume into segments corresponding to each electrode using the “SplitIsland” filter and (3) adding a point (3D space) at the centroid of each segmented electrode using “SegmentStatistics” [10]. After extracting electrode locations from the CT image, prescribed displacements are computed using our automated procedure, which comprises the following steps (Fig. 6): (1) projection of electrodes on the undeformed brain surface extracted from pre-operative MRI, (2) creating the electrode sheet model (representing the silastic substrate of the electrocorticography electrode grid placed on the brain surface in epilepsy surgery) using the projected electrodes, (3) selecting brain surface nodes (known as loaded nodes) under the electrode sheet model and (4) computing prescribed displacements.

In step 1, we projected the extracted electrodes onto the surface of the brain extracted from the pre-operative MRI using our “MarkUpsToDistance” module. This module determines the points corresponding to the location of electrodes (referred to as projected electrodes) in the undeformed (pre-operative) brain configuration. The module uses the “ImplicitPolyDataDistance” method of the Visualization Toolkit (VTK) [21] to compute the distance for each of the electrodes identified in the post-operative CT to its corresponding nearest triangle on the undeformed brain surface. We created projected electrode locations at the centroids of the identified nearest triangles on the undeformed brain surface (extracted from pre-operative MRI). In step 2, we used these projected electrode locations to create an electrode sheet model by means of the PolyData algorithm [22], implemented in our “SheetFromPoints” module. We uniformly triangulated the electrode surface sheet model using the PyACVD software library (https://github.com/pyvista/pyacvd) implemented in our module “SurfaceTriangulation”. In step 3, we used the vertices of each triangle of the electrode sheet model to select the corresponding brain surface cells (triangles) in the undeformed brain surface model using our “NodeSelector” module. We used the selected brain surface cells to select the corresponding nodes (vertices) of these selected brain surface cells. We applied prescribed displacements on the selected nodes of the selected triangles. In step 4, to compute prescribed displacements, the original and projected electrode coordinates were used as an input to the “ScatteredTransform” [23] module to compute a B-Spline transform. We applied the computed B-Spline transform to the undeformed brain surface nodes located under the electrode sheet to determine the position of the corresponding nodes in the deformed (due to electrode implantation) brain geometry. We computed the prescribed displacements as the difference between the coordinates of the corresponding brain surface nodes in undeformed and deformed brain geometry. The prescribed displacements were applied using a smooth (3-4-5 polynomial) loading curve [24].

In tumour resection-induced brain shift scenarios, we consider the load as a gravity force and calculate the internal forces acting at the interface nodes between the tumour and nearby healthy brain tissues. Once the tumour is removed from the biomechanical model, the reaction forces are applied in the opposite direction to the interface nodes. This approach allows us to simulate the deformation of the brain tissue caused by the tumour and its subsequent removal during surgery.

#### Assignment of material properties using fuzzy tissue classification

In patient-specific computational biomechanics modelling, the material properties are typically assigned using image segmentation [25, 26], where each image voxel is assigned to a specific brain tissue class using semi-automatic procedures. However, this process is time-consuming and clinically incompatible [25]. We used fuzzy tissue classification [27] to automatically assign material properties of intra-cranial constituents to integration points within the problem domain. To assign material properties to brain tissues, we used our “FuzzyClassification” module that takes a brain mask (see generated brain mask in Sect. "Computational grid generator"), pre-operative MRI and tumour mask (if present) as inputs and produces fuzzy classified brain tissue classes, which are used by our “MaterialPropertiesAssignment” module to automatically assign material properties to integration points corresponding to brain constituents.

For all nine case studies, we used a mass density of 1000 kg/m^3^ for all tissue types. Craniotomy and electrode placement-induced brain shift simulations correspond to a subclass of problems known as “displacement–zero traction” problems, where the load is defined by prescribing the displacement on the boundary, and we do not know anything about the deformation field within the analysed continuum. Problems of this type depend very weakly on the material properties and material model. Therefore, we used a neo-Hookean constitutive model, with initial Young’s modulus *E*, and initial Poisson’s ratio *ν*, listed in Table 3 [26].

**Table 3:** Neo-Hookean material model parameters for each tissue type in the biomechanical brain model used for computing craniotomy and electrode placement-induced brain shift

Tissue type	*ρ* (kg/m3)	*E* (Pa)	*ν*
Parenchyma	1000	3000	0.49
Tumour	1000	9000	0.49
Ventricle	1000	10	0.1

Poisson’s ratio is a mechanical property that describes the compressibility of a material. A low Poisson’s ratio suggests strong compressibility, whereas a high Poisson’s ratio of 0.5 indicates that the material is fully incompressible. We consider the parenchyma of the brain to be a nearly incompressible structure [28]. In the tumour resection-induced brain shift, since the load is due to gravity and traction forces, the computed deformations depend on the tissue “stiffness” as determined by the material properties and material model. Therefore, an Ogden constitutive model is used, with shear modulus *µ*, initial Poisson’s ratio *ν* and material parameter *α*, listed in Table 4 [29], because it adequately accounts for the brain tissue material properties under both tension and compression. The shear modulus for the tumour was assigned a value three times larger than that of healthy brain tissue [28].

**Table 4:** Ogden material model parameters for different tissue types (CSF, parenchyma, tumour) in the biomechanical brain model used for computing brain shift due to tumour resection. Note that for α = 2 the Ogden model is similar to the neo-Hookean model

Tissue type	*µ* (Pa)	*ν*	*α*
Parenchyma	842	0.49	− 4.7
Tumour	2526	0.49	− 4.7
CSF (case 4)	4.54	0.1	2
CSF (cases 5, 6, 7)	4.54	0.49	2

### Model solution

To compute brain deformations using MTLED, we developed the “MTLEDSolver” module that uses the meshless total Lagrangian explicit dynamics (MTLED) algorithms [5]. The module predicts intra-operative deformations and generates a solution in the form of a deformed brain biomechanical model. The MTLED solution algorithm is described in detail in our previous studies [5, 7]. MTLED solves the weak form of the elasticity equations and can be used with different shape functions, including moving least squares (MLS) [5], modified moving least squares (MMLS) [7] and interpolating modified moving least squares (IMMLS) [30]. The methodology for computing brain deformations has been extensively validated in our previous studies [7, 8, 31]. We use IMMLS shape functions [30] as they accurately enforce the essential boundary conditions and provide robust computations for large deformations and strains.

### Image warping

To perform image warping, we extracted undeformed and predicted deformed brain model nodal coordinates and used the “ScatteredTransform” module [23] to compute a B-Spline transform which is used to warp the pre-operative MRI to obtain the predicted intra-operative MRI.

## Results

In this section, we apply our framework to solve three neurosurgical brain shift scenarios: craniotomy, tumour resection and electrode placement-induced brain shift.

### Methods for evaluating predicted intra-operative deformations by our framework

We evaluated our framework qualitatively and quantitatively by analysing the predicted intra-operative MRI and the actual intra-operative MRI of nine patients. For qualitative evaluation, we visually compared the predicted brain contour with the actual intra-operative brain contour. For quantitative evaluation, following [26, 32], we use the Hausdorff distance (HD) to objectively measure the differences between the ventricle surfaces of the brain predicted by our framework using the MTLED algorithm and the ventricle surfaces obtained by segmentation of the actual intra-operative MRI.

The purpose of this study is to create a framework for automating the workflow for predicting intra-operative brain deformations rather than to conduct evaluation of the framework performance using patient cohort sufficiently large for comprehensive statistical analysis. We have made our open-source framework freely available through GitHub, which opens avenues for other research groups to use the framework and conduct its independent evaluation. In this study, we demonstrate the application of the framework by predicting intra-operative deformations in nine patients undergoing neurosurgical procedures. Given this relatively small cohort size, we conduct only rudimentary statistical analysis of the results by reporting the average and standard deviation of the percentage of successfully registered points/nodes (i.e. the nodes for which the registration error is lower than twice the in-plane resolution of the intra-operative image).

### Craniotomy-induced brain shift

#### Qualitative evaluation

**Fig. 7:**
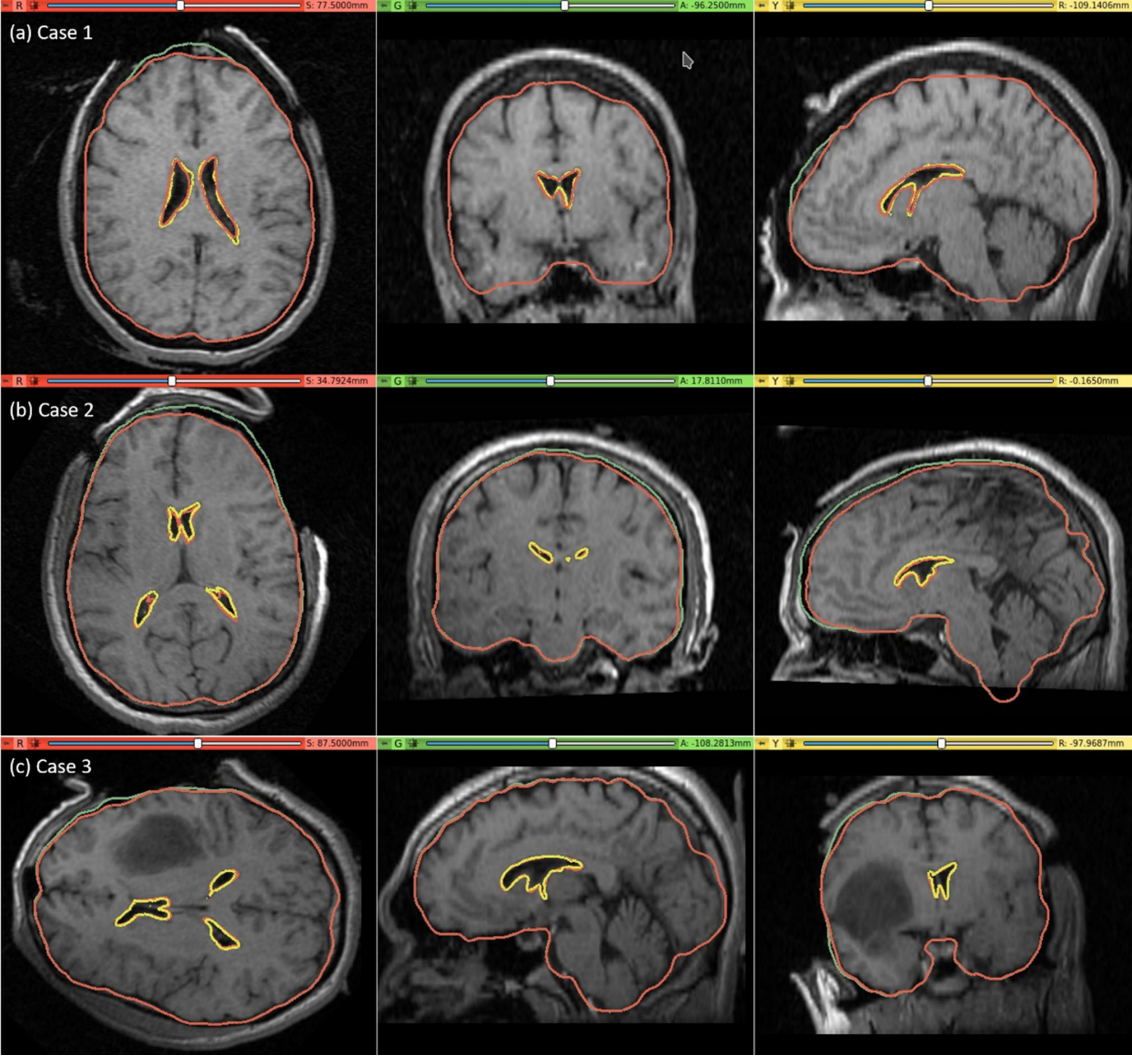
Intra-operative MRI overlaid with contours (red) of the deformed ventricle and brain extracted from the predicted intra-operative MRI which is obtained by warping the pre-operative MRI with the computed transform. Comparison of the brain contours (red) from the predicted intra-operative MRI along with the brain contours (green) extracted from the pre-operative MRI. Predicted red ventricle contours and intra-operative ventricle contours in yellow

The pre-operative MRI was warped to obtain the predicted intra-operative MRI using the B-Spline transformation described in Sect. "Image warping" such that it corresponds to the actual intra-operative anatomy of the brain. We visually compared the brain contour predicted by our framework (from a warped pre-operative MRI) with the actual intra-operative MRI. The ventricle contours predicted by our automated framework (Fig. 7) for case studies 1, 2 and 3 are very close to the actual intra-operative ventricle contours.

#### Quantitative evaluation

**Fig. 8:**
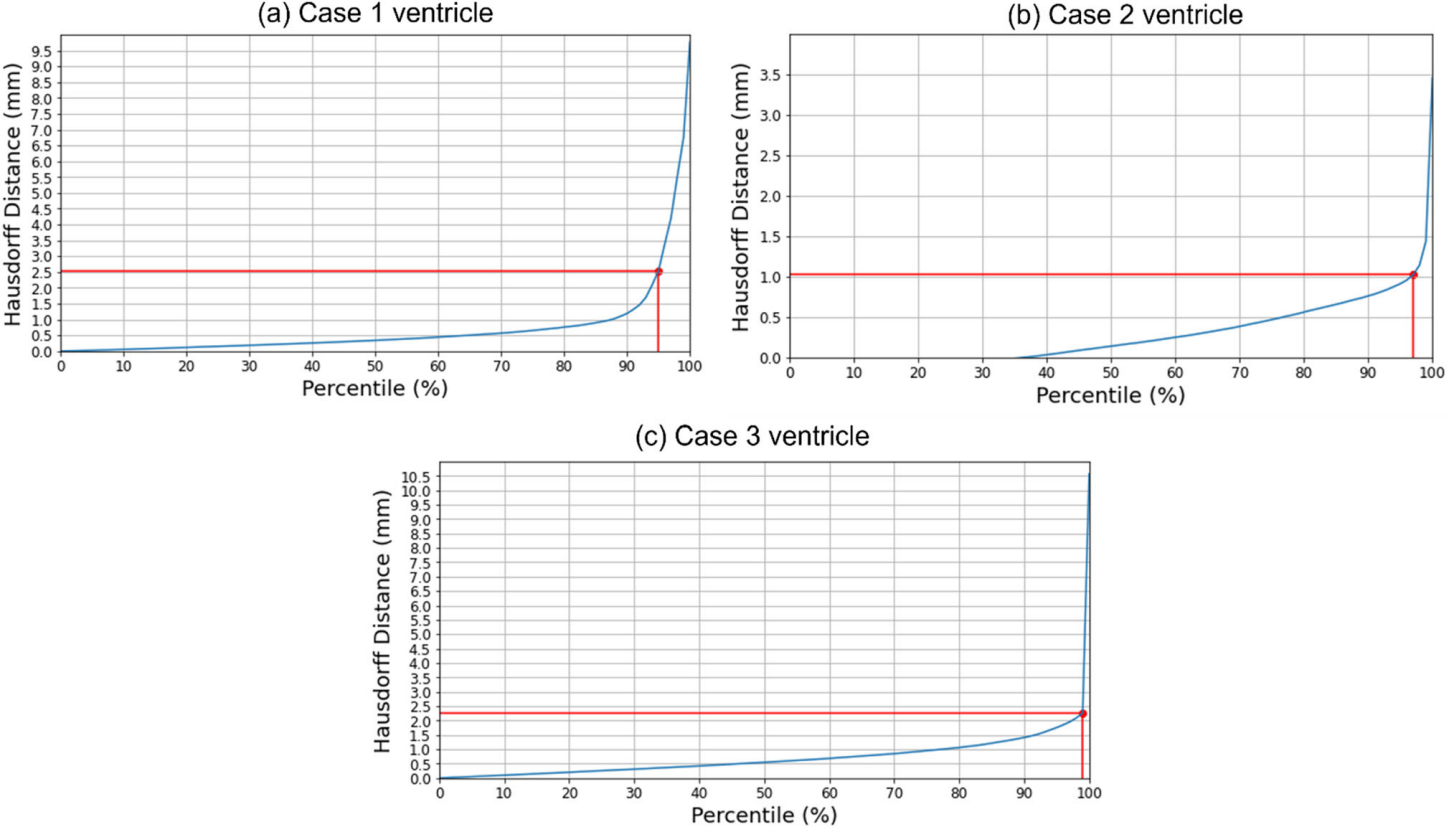
Hausdorff distance between predicted and actual intra-operative ventricles at different percentiles. The in-plane resolution of the intra-operative image for all patients is indicated by the red line. **a** For patient 1, the acceptable registration error is 2.5 mm, which corresponds to 96-percentile HD, **b** for patient 2, the acceptable registration error is 1 mm, which corresponds to 98-percentile HD, and **c** for patient 3, the acceptable registration error is 2.5 mm, corresponds to 99-percentile HD

We used the 95th, 75th, 50th and 25th percentile HD to measure the similarities between the actual ventricle surfaces (obtained from segmentation of actual pre-/post-operative MRIs) and the predicted ventricle surfaces (obtained from the segmentation of the predicted MRIs, see Table 5). The image resolution limits the precision of neurosurgical image guidance. Therefore, registration is considered successful if the 95% HD is lower than twice the actual in-plane resolution of the intra-operative MRI (2.5 mm, 1 mm and 2.5 mm for case studies 1, 2 and 3, respectively). For case studies 1, 2 and 3, about 96%, 98% and 99% of the nodes on the ventricle surfaces, respectively, were successfully registered (Fig. 8). The results obtained using our automated framework are very close to those reported in our previous studies [8, 26] (see Table 5). The mean 95th percentile Hausdorff distance between the ventricle surfaces for the three craniotomy-induced brain shift case studies is 1.9 mm with a standard deviation (SD) of 0.464 mm. This means that the overall agreement between the ventricles is reasonably good, with most of the points falling within one standard deviation of the mean.

**Table 5:** Quantitative evaluation for craniotomy-induced brain shift. 95th, 75th, 50th and 25th percentile (millimetres) of HD between the predicted and actual ventricle surfaces. The 95% HD was utilised as the measure of registration error. The results are compared to finite element and MTLED results from our previous studies [8, 26]

Case	H95 (mm)	H75 (mm)	H50 (mm)	H25 (mm)
1	1.7	0.9	0.5	0.2
1 [32]	1.3	0.6	0.4	0.3
2	1.5	0.9	0.5	0.2
2 [32]	2.8	1.2	0.8	0.4
2 [10]	1.4	N/A	N/A	N/A
3	2.5	0.6	0.3	0.2
3 [32]	1.9	1.1	0.6	0.4

### Tumour resection-induced brain shift

#### Qualitative evaluation

**Fig. 9:**
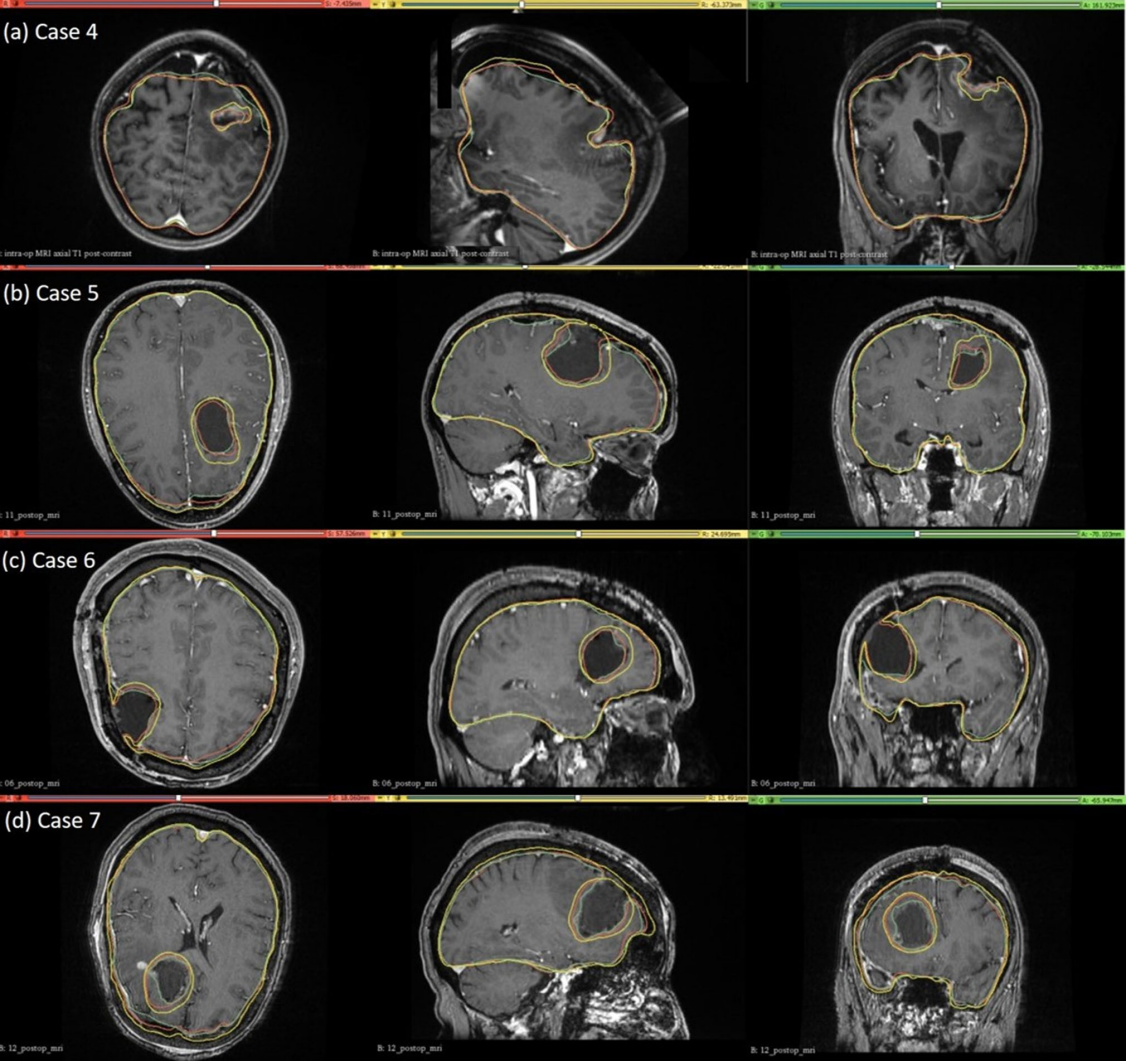
Intra-operative (case 4) and post-operative (cases 5, 6 and 7) MRIs overlaid with brain and tumour contours. The yellow in the tumour region denotes the pre-operative tumour cavity, whereas the green denotes the true intra-operative tumour cavity in case 4 and the post-operative tumour cavity in cases 5, 6 and 7. For the brain contours, yellow denotes pre-operative brain contour, green represents intra-operative brain contour for case 4 and post-operative brain contour for cases 5, 6 and 7, and red denotes predicted brain contour

For case study 4, the predicted intra-operative brain contour extracted from the predicted intra-operative MRIs was compared to the actual intra-operative MRI brain contour (Fig. 9). Likewise, we qualitatively evaluated our framework’s predicted contours of the brain parenchyma extracted from predicted post-operative MRIs for case studies 5, 6 and 7 to the actual brain contours extracted from the actual post-operative MRI (Fig. 9). The predicted maximum displacement observed in case studies 4, 5, 6 and 7, was 11 mm, 7 mm, 7.2 mm and 6.5 mm, respectively.

#### Quantitative evaluation

**Fig. 10:**
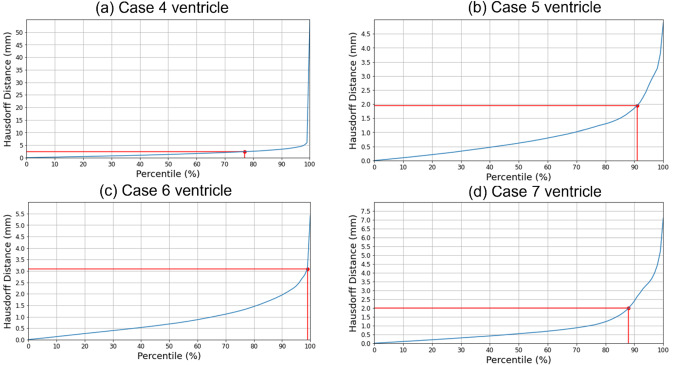
Hausdorff distance between predicted and actual intra-operative ventricles at different percentiles. The in-plane resolution of the intra-operative image for all patients is indicated by the red line. **a** For patient 4, the acceptable registration error is 2.4 mm, corresponding to 78-percentile HD, **b** for patient 5, the acceptable registration error is 2 mm, corresponding to 92-percentile HD, **c** for patient 6, the acceptable registration error is 4 mm, corresponding to 99-percentile HD, and **d** for patient 7, the acceptable registration error is 2 mm, corresponding to 89-percentile HD

We used the 95th, 75th, 50th and 25th percentile HD to measure the similarities between the actual ventricle and the predicted ventricle surfaces (Table 6). Registration is considered successful if the 95% HD is lower than twice the actual in-plane resolution of the intra-operative MRI (2.4 mm, 2 mm, 4 mm and 2 mm for case studies 4, 5, 6 and 7, respectively). For case study 4, about 77% of the nodes on the ventricle surfaces were successfully registered. For case studies 5, 6 and 7, about 92%, 99% and 89% of the nodes on the ventricle surfaces, respectively, were successfully registered (Fig. 10). The mean 95th percentile Hausdorff distance between the ventricle surfaces for the four tumour resection-induced brain shift case studies is 3.1 mm (SD = 0.842 mm). This shows that the overall agreement between the ventricles is reasonably good, with most points falling within one standard deviation of the mean. However, the slightly higher values of mean HD indicate that the tumour resection-induced brain shift is more difficult to compensate for than craniotomy-induced brain shift.

**Table 6:** Quantitative evaluation for tumour resection-induced brain shift. 95th, 75th, 50th and 25th percentile (millimetres) of HD between the predicted and actual ventricle surfaces

Case	H95 (mm)	H75 (mm)	H50 (mm)	H25 (mm)
4	4.10	2.29	1.27	0.58
5	2.67	1.15	0.62	0.27
6	2.34	1.26	0.68	0.33
7	3.44	1.01	0.54	0.25

### Electrode placement-induced brain shift

**Fig. 11:**
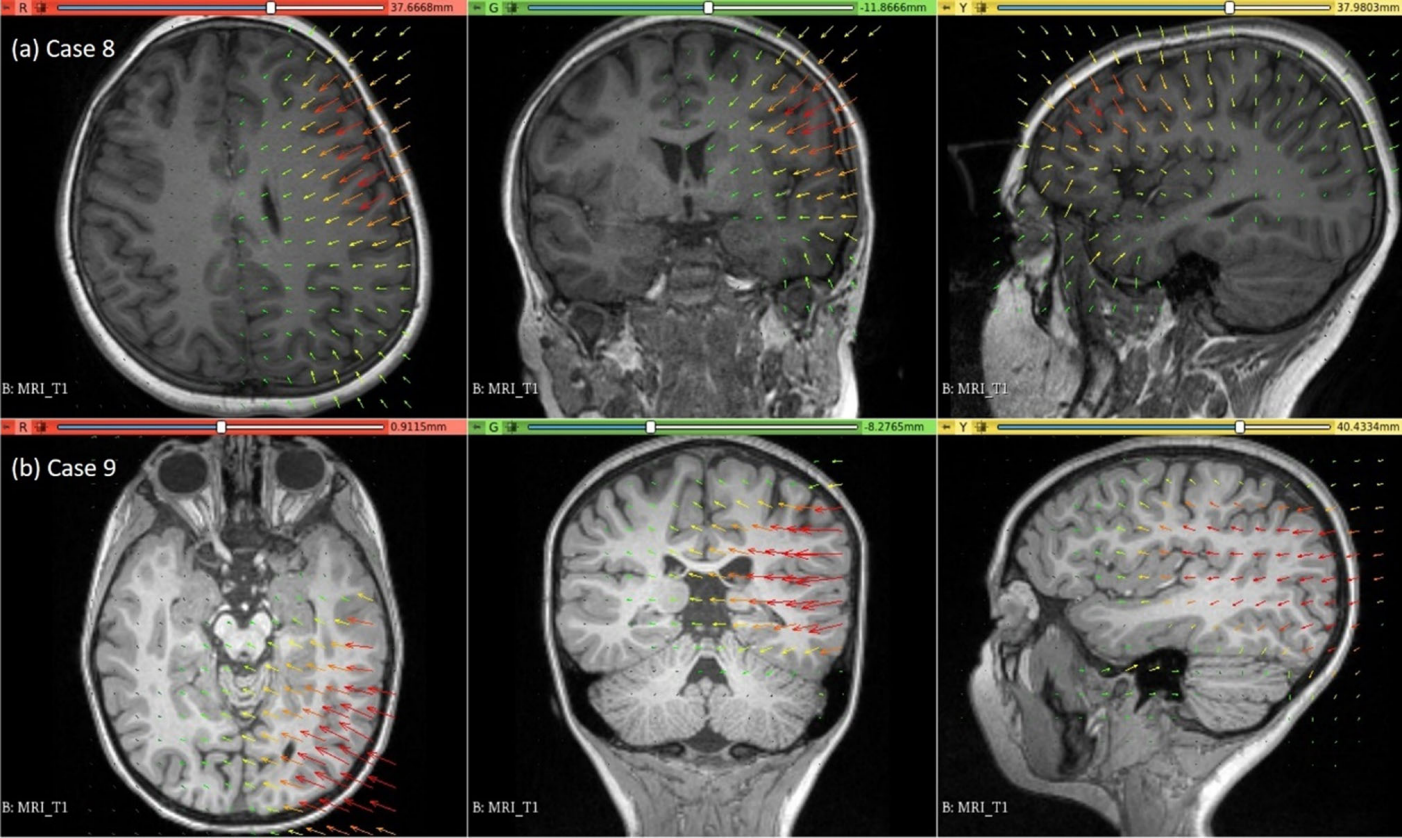
Visualisation of deformation field computed using our automated framework for cases 8 and 9

**Fig. 12:**
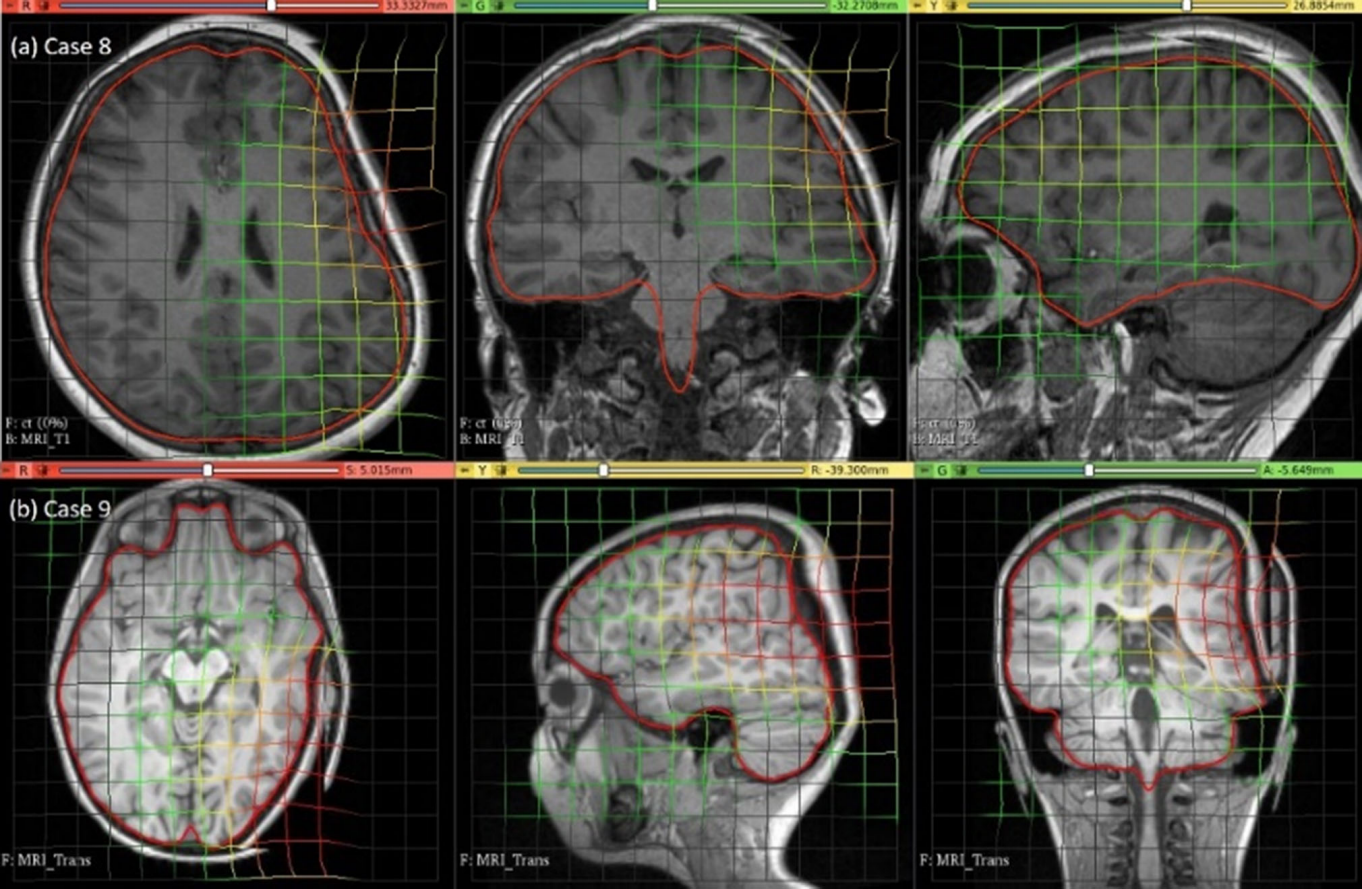
Visualisation of pre-operative image transformation for cases 8 and 9 using deformation field from Fig. 10a and b, respectively, and predicted deformed brain model surface (red line) overlaid with predicted image

**Fig. 13:**
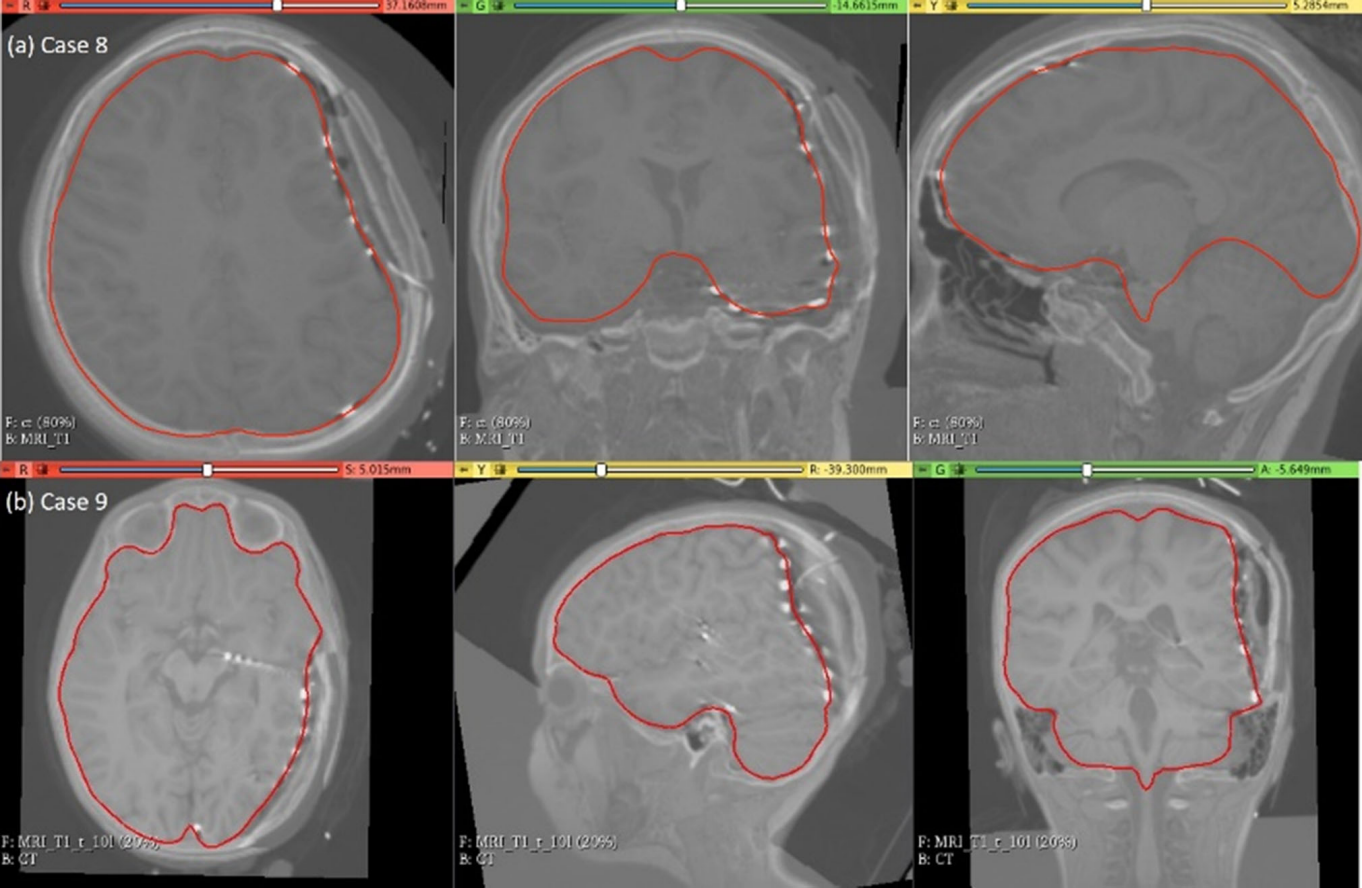
Visualisation of predicted intra-operative MRIs for case 8 and case 9 registered on CT with implanted intra-cranial electrodes along with predicted deformed brain model surface (red line)

The predicted maximum displacement in case studies 8 and 9 was 11.7 mm and 21.5 mm, respectively. Using the deformation field predicted by our framework, we warped the pre-operative MRI to obtain the corresponding brain configuration with electrodes implanted. We used the “Scattered Transform” module [23] to obtain the transform for image warping. Figure 11 shows the computed deformation field of case 8 (Fig. 11a) and case 9 (Fig. 11b). Figure 11 shows the transforms used to warp the pre-operative MRI of case 8 (Fig. 12a) and case 9 (Fig. 12b). The results of the registration are shown in Fig. 13a for case 8 and in Fig. 13b for case 9.

### Computational efficiency

The simulations were performed on an HP Pro-Book laptop with an Intel Core i7 (2.7 GHz) processor and 8 GB physical memory. The time required to automatically generate a patient-specific brain biomechanical model using our framework was less than 3 min for each case. However, in tumour resection, there is an additional step of the construction of a brain model with a tumour cavity, which takes approximately 20 min. The solution to the biomechanical models ranged from 13 to 23 min.

## Discussion and conclusion

We developed a framework to automatically generate a brain biomechanical model and compute intra-operative brain deformations. Our framework, SlicerCBM, is implemented as an extension in 3D Slicer, freely open-source software, and contains modules that can be used in combination to solve three neurosurgical brain shift scenarios: craniotomy, tumour resection and electrode placement-induced brain shift. The main modules of the framework are “ComputationalGridGenerator” to generate a patient-specific computational grid, “CranGenerator” to create a craniotomy model, “SurfaceTriangulation” to generate a uniformly triangulated surface (craniotomy and electrode sheet), “ElectrodeToMarkups” to extract the original electrode locations from CT, “MarkUpsToDistance” to create the projected electrode locations on the undeformed brain surface, “SheetFromPoints” to generate an electrode sheet model, “NodeSelector” to select the exposed brain surface due to a neurosurgical procedure, “DisplacementLoading” to compute loading, “SkullGenerator” to define the boundary conditions, “FuzzyClassification” and “MaterialPropertiesAssignment” to assign material properties to intra-cranial constituents and “MTLEDSolver” to compute brain deformations. These modules uniquely combine various algorithms working behind 3D Slicer modules and open-source software libraries. The “MTLEDSolver” module integrates our MTLED algorithm to provide an interface to this robust and efficient solution algorithm.

We evaluated the accuracy of our framework by performing nine simulations belonging to three neurosurgical brain shift scenarios. For craniotomy and tumour resection, the actual ventricle contours (yellow) and the ventricle contours predicted by our framework (red) show good similarity (Figs. 7 and 9). The 95% HD for ventricles surfaces for all case studies is less than two times the original in-plane resolution of the intra-operative MRI, which confirms successful registration. The 95% HD of the ventricle surfaces between the predicted and actual intra-operative MRIs for case study 2, between the results produced by our automated framework and the results obtained in our previous studies [8, 26], is less than 0.1 mm. The results obtained using our automated framework are very close to those reported in our previous studies [8, 26].

Our framework needs further verification against large cohort patient studies. Furthermore, the quantitative evaluation of displacements for electrode placement-induced brain shift was not possible due to the lack of intra-operative MRI data, as MRIs with electrodes implanted within the brain cannot be obtained for patient safety reasons. Our framework has significant potential for clinical applications. Qualitative and quantitative comparisons of ventricle surfaces in predicted and intra-operative MRIs for craniotomy and tumour resection-induced brain shift, and qualitative comparisons of brain contours for electrode placement-induced brain shift, lead us to conclude that the results are accurate enough to be useful in clinical applications because the accuracy of the results that we obtained for all case studies is within the limits typically required in image-guided surgery [33].

## Data Availability

The software developed as part of this study is bundled as the SlicerCBM extension for 3D Slicer and is freely available from our GitHub repository (https://github.com/SlicerCBM/SlicerCBM).
